# Kyphoplasty Restores the Global Sagittal Balance of the Spine Independently from Pain Reduction

**DOI:** 10.1038/s41598-020-65798-0

**Published:** 2020-06-01

**Authors:** Matthias Pumberger, Florian Schitz, Justus Bürger, Friederike Schömig, Michael Putzier, Yannick Palmowski

**Affiliations:** 1 Charité – Universitätsmedizin Berlin, corporate member of Freie Universität Berlin, Humboldt-Universität zu Berlin, and Berlin Institute of Health, Center for Musculoskeletal Surgery, Chariteplatz 1, 10117 Berlin, Germany; 2 Medical University of Vienna, Spitalgasse 23, Vienna, 1090 Austria

**Keywords:** Outcomes research, Fracture repair

## Abstract

Kyphoplasty is the standard surgical treatment of vertebral compression fractures. We aimed to clarify the influence of kyphoplasty on the sagittal profile as well as the relation between posture improvement and pain relief. For this purpose, we evaluated various radiological parameters of the sagittal profile on whole spine standing radiographs of 73 Patients with a single vertebral fracture treated by kyphoplasty. The key outcome was the postoperative change of the sagittal vertical axis (SVA). Additionally, clinical parameters including pain scores on visual analogue scale (VAS) and use of analgesics were obtained from medical records. Pre- and postoperative radiological as well as clinical parameters were compared. Additionally, the correlation between changes of SVA and changes of local kyphotic angle (LKA) or VAS was examined. The clinical parameters as well as various radiographic parameters (SVA, LKA, Gardner, Cobb) improved significantly postoperatively. The improvement of SVA correlated significantly with the correction of the LKA but not with postoperative pain relief. We conclude that kyphoplasty helps to restore the global sagittal balance of the spine after vertebral fractures. The correction of the sagittal profile seems to depend on the correction of the local kyphotic angle but does not correlate with postoperative pain relief.

## Introduction

Osteoporosis is a major cause of fractures in elderly patients, leading to more than 8.9 million fractures per year worldwide^1^. The most common type of osteoporotic fractures are vertebral fractures^2^. With around 1 million incident vertebral fractures per year in Europe alone, causing costs of estimated €377 million, they constitute a major therapeutic and socioeconomic challenge in aging societies^3,4^.

Despite their high relevance, there is still no clear consensus regarding the optimal treatment. As osteoporotic fractures are mainly stable compression fractures, both conservative and operative treatment options can be considered^5^. Regarding operative treatment options, vertebroplasty and kyphoplasty are the most widely used techniques. While vertebroplasty mainly aims at quick pain relief by stabilizing the fractured vertebra with bone cement, kyphoplasty additionally offers the possibility to correct fracture-related deformities by inflating a balloon in the fractured vertebral body before filling it with bone cement. The efficacy of kyphoplasty for short-term pain relief is well documented and widely accepted^6,7^. However, the advantage of kyphoplasty over conservative therapy regarding pain relief seems to diminish over time^8^. Additionally, there is so far no study comparing the pain relief of kyphoplasty to the placebo-effect of a sham procedure^8^. Therefore, the key factors for the evaluation of the long-term benefit/risk ratio of kyphoplasty seem to be the restoration of lost vertebral body height and the correction of fracture-related spinal malposition.

Such spinal malposition can have various implications. In many cases, vertebral compression fractures result in a kyphosis of the affected vertebral body. The effects are not restricted to the area of the fracture but rather affect the spine as a whole. Any increase of kyphosis will inevitably shift the center of gravity ventrally, thereby changing the weight force distribution of the remaining vertebral bodies. This explains why hyperkyphosis is not only a common result of vertebral compression fractures, but also in itself an independent risk factor for future fractures^9^. Also, thoracic kyphosis and lumbar lordosis are positively correlated, so that thoracic hyperkyphosis may result in a compensatory increase in lumbar lordosis^10^. A higher lumbar lordosis has been reported to be associated with lower back pain^11^.

There is consistent evidence that kyphoplasty can achieve (partial) height restoration of the fractured vertebrae^12–14^. Yet, the effect of these local changes on the global sagittal profile of the spine has been hardly examined so far. Only few studies have been conducted in small populations and came to conflicting conclusions^15–18^. This lack of knowledge about the potential long-term benefits constitutes an important limitation when trying to determine the appropriate indication of the kyphoplasty. As a result, most guidelines remain rather vague and recommend the intervention only after a futile conservative treatment attempt^5^. The potential long-term advantages of a restored sagittal profile are not taken into account as no reliable evidence to this effect is available.

Therefore, more detailed knowledge about the effect of kyphoplasty on the sagittal profile of the spine is urgently needed for a differentiated evaluation of the long-term risk/benefit ratio and to thereby allow for more precise treatment recommendations for vertebral compression fractures.

In the present study, we hypothesized that percutaneous balloon kyphoplasty has a significant impact on parameters of the sagittal profile of the spine and conducted a retrospective analysis of patients treated in our department.

## Materials and methods

### Patients

For this retrospective cohort study we included consecutive patients that received balloon kyphoplasty of a single vertebra due to a vertebral compression fracture between January 2014 and December 2018. Kyphoplasty was performed in a standardized manner with the patients being placed in a lordosing prone position and using a biportal technique. Only patients with pre- and postoperative upright whole spine radiographs obtained within not more than 30 days before and after the operation were eligible. All patients had undergone a futile conservative treatment attempt prior to the operation. Patients were excluded if they had received additional spinal operations (e.g. instrumented spinal fusion) or sustained new vertebral fractures between pre- and postoperative radiographs. Patients were also excluded if they had undergone a prior instrumented spinal fusion including L5/S1 as this would not have allowed a reliable measurement of spinopelvic parameters.

### Data collection and image analysis

**Figure 1:**
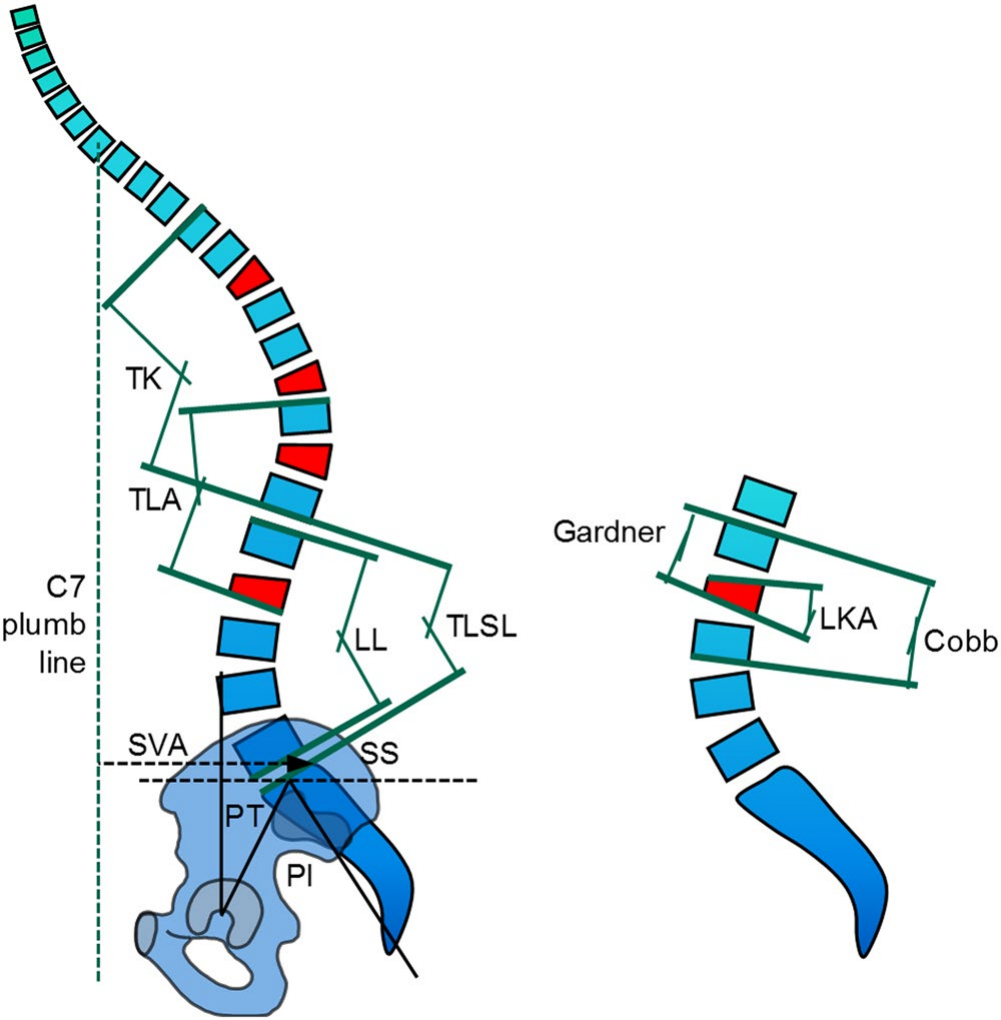
Global and Local Radiographic Parameters (Cobb: Cobb angle, Gardner: Gardner angle, LKA: locaFigurephotic angle, LL: lumbar lordosis, PI: pelvic incidence, PT: pelvic tilt, SS: sacral slope, SVA: sagittal vertical alignment, TK: thoracic kyphosis, TLA: thoraco-lumbar alignment, TLSL: thoraco-lumbo-sacral lordosis). Figure adapted from^19^ with kind permission from the author and *Deutscher Ärzteverlag*.

Pre- and postoperative lateral whole spine standing radiographs were evaluated regarding radiologic parameters of fracture morphology and global sagittal profile (Fig. 1). Image analysis was carried out in a standardized manner according to previously published methods using Centricity DICOM Viewer (GE Healthcare, Buckinghamshire, United Kingdom)^19^. The mean values from independent measurements by two reviewers (a spinal surgeon experienced with the measurements of radiologic spinal parameters and an associate researcher who received prior training) were used for statistical analysis in order to minimize the risk of systematic bias. The following parameters were measured: angle between cephalad and caudad endplate of the fractured vertebra (local kyphotic angle, LKA); angle between the cephalad endplate of the vertebra proximally adjacent to the fracture and the caudad endplate of the fractured vertebra (Gardner angle, GA); angle between the cephalad endplate of the vertebra proximally adjacent to the fracture and the caudad endplate of the vertebra distally adjacent to the fracture (Cobb angle, CA); distance between the C7 plumb line and the rear edge of S1 (sagittal vertical axis, SVA); angle between the sacral endplate and a horizontal reference line (sacral slope, SS); angle formed by a line connecting the centre of the hip with the centre of the sacral endplate and a vertical reference line (pelvic tilt, PT); angle between the cephalad endplate of L1 and the caudad endplate of L5 (lumbar lordosis, LL); angle between the caudad endplate of Th12 and the sacral endplate (thoracolumbosacral lordosis, TLSL); angle between the cephalad endplate of Th10 and the caudad endplate of L3 (thoracolumbal alignment, TLA); and the angle between the cephalad endplate of Th4 and the caudad endplate of Th12 (thoracal kyphosis, TK). The key outcome was defined as the postoperative change in SVA.

In addition, clinical data including the presumed time of the fracture (defined as the moment of symptom onset), pre- and postoperative pain at rest or in motion (on visual analog scale, VAS) and need of analgesics (according to WHO Analgesic Ladder) were obtained from the medical records.

### Statistical analysis

Statistical analysis was performed using SPSS software version 23 (IBM, New York, USA). Mean and standard deviation were calculated for descriptive patient characteristics. Pre- and postoperative values for VAS scores (at rest and in motion) and radiographic measurements were compared using Student's t-test for paired samples. Pre- and postoperative need for analgesics according to the WHO pain ladder were tested for significant differences using Wilcoxon signed-rank test. The difference between pre- and postoperative radiographic parameters was tested for correlation with pre- and postoperative differences in VAS score and LKA by calculating Pearson's correlation coefficient.

### Ethics approval

The study was approved by the institutional review board of Charité – Universitätsmedizin Berlin (EA1/188/18). The study was carried out in accordance with relevant guidelines and regulations.

## Results

### Study population

A total of 73 patients (average age 70 years) were identified that matched the inclusion criteria (Table 1). All included patients only had a single fracture, with 33 (45%) in the thoracic and 40 (55%) in the lumbar spine. 8 fractures were due to an adequate trauma and 65 fractures were not caused by an adequate trauma (atraumatic or due to low-energy trauma, i.e. a fall from standing height or below). Osteoporotic treatment with bisphosphonates was preoperatively documented in 14 (19%) patients. The average time between fracture and surgical intervention was 4.7 weeks, ranging from 1 to 40 weeks. The average time between preoperative X-ray and operation was 5.1 (±4.5) days, the average time between operation and postoperative X-ray was 2.8 (±3.3) days.

**Table 1:** Characteristics of the study population.

Patients (n)	73
Male	26
Female	47
Age (years; SD)	70 (10.9)
BMI (kg/m²; SD)	25.13 (4.18)
Level (n)	
**Thoracic Spine**	**33**
6	1
7	1
8	7
9	5
10	1
11	7
12	11
**Lumbar Spine**	**40**
1	18
2	9
3	7
4	4
5	2
Osteoporotic Medication (n)	
Vitamin D	28
Bisphosphonates	14

### Pain scores

Patients showed a significant decrease in pain at rest and in motion (Table 2). Furthermore, comparison between pre- and postoperative need of analgesics according to the WHO pain ladder revealed a significant decrease of pain medication use after kyphoplasty (Z = −4.01, p <0.001).

**Table 2:** Pre- and postoperative pain (VAS scores).

	Preoperative	Postoperative	Difference	P-Value
**VAS Score (SD)**				
at rest	3.7 (2.8)	1.7 (1.7)	2 (2.8)	<0.001
in motion	5.1 (2.5)	2.8 (2)	2.4 (2.8)	<0.001

### Radiographic measurements

The detailed pre- and post-operative radiographic measurements are depicted in Table 3. In summary, the SVA significantly decreased post-operatively. Furthermore, LKA, Gardner and Cobb angle changed significantly. However, pelvic parameters as the PT did not change significantly. Inter-rater reliability was generally high with intraclass correlation coefficients ranging from 0.841 (postoperative LKA) to 0.995 (preoperative SVA).

**Table 3:** Difference between pre- and post-operative radiographic measurements.

	Mean preoperative (SD)	Preoperative ICC	Mean postoperative (SD)	Postoperative ICC	Difference (SD)	p-value*
SVA [mm]	50.79 (37.96)	0.995	40.5 (34.48)	0.994	10.29 (30.11)	0.038
LL [°]	33.21 (12.94)	0.97	34.71 (12.05)	0.974	−1.5 (4.97)	n.s.
TK [°]	46.81 (13.99)	0.978	46.08 (13.46)	0.977	0.72 (7.2)	n.s.
SS [°]	35.92 (9.19)	0.946	35.91 (9.32)	0.949	0.01 (5.12)	n.s.
PT [°]	20.66 (7.96)	0.972	20.85 (7.31)	0.893	−0.19 (4.71)	n.s.
PI [°]	56.07 (11.17)	0.962	55.56 (11.02)	0.911	0.51 (4.53)	n.s.
TLSL [°]	51.59 (14.24)	0.973	52.86 (12.92)	0.96	−1.27 (6.53)	n.s.
TLA [°]	−11.58 (17.2)	0.974	−10.25 (15.5)	0.986	−1.34 (8.51)	n.s.
LKA [°]	14.14 (7.6)	0.942	9.85 (6.54)	0.841	4.29 (3.86)	<0.001
Gardner [°]	15.99 (12.36)	0.967	12.51 (11.27)	0.858	3.48 (5.32)	<0.001
Cobb [°]	15.28 (15.42)	0.961	10.97 (16.64)	0.935	4.31 (7.1)	<0.001

Cobb: Cobb angle, Gardner: Gardner angle, ICC: intraclass correlation coefficient, LKA: local kyphotic angle, LL: lumbar lordosis, PI: pelvic incidence, PT: pelvic tilt, SS: sacral slope, SVA: sagittal vertical alignment, TK: thoracic kyphosis, TLA: thoraco-lumbar alignment, TLSL: thoraco-lumbo-sacral lordosis.

*p-values adjusted for multiple testing according to Bonferroni-Holm

### Correlation of SVA with clinical and radiographic parameters

The correlation analysis revealed a significant correlation between pre-/postoperative difference in SVA and pre-/postoperative difference in LKA (Table 4). However, the change in SVA did not correlate to postoperative pain reduction.

**Table 4:** Correlations of pre-/postoperative changes in sagittal vertical axis (SVA) with changes in local kyphotic angle (LKA) and pain according to visual analogue scale (VAS) at rest and in motion.

		Difference in LKA	Difference in VAS (at rest)	Difference in VAS (in motion)
Difference in SVA	Pearson correlation coefficient	0.305	−0.084	−0.080
	p-value	0.009	n.s.	n.s.

### Subgroup analyses of patients with positive sagittal imbalance

31 patients presented with a positive sagittal imbalance (defined as SVA > 50 mm) preoperatively. In the subgroup analysis of these patients, the mean SVA decreased from 84.36 (±32.48) to 58.61 (±33.3). The mean increase in SVA was 25.75 (±27.43) (p < 0.001). 25 patients still had a positive sagittal imbalance postoperatively.

## Discussion

In the present work, we have conducted the largest study so far regarding the influence of kyphoplasty on the sagittal profile of the spine. Our results show a significant improvement of the SVA in patients with vertebral fractures after kyphoplasty. This result is of wide-ranging significance as it suggests a long-term benefit of kyphoplasty over conservative treatment. The results are in concordance with those from a smaller previous study, which included 21 patients^17^. However, two other previous studies did not find a significant change in SVA after kyphoplasty. One reason for this might be the timed passed since the occurrence of the fracture. In our study, the average time between fracture and kyphoplasty was 1.2 months. In both studies that did not find a change in SVA postoperatively, the time from injury to kyphoplasty was either much higher with an average of 3.4 months^15^ or not reported at all^16^. In a reverse conclusion, a long conservative treatment attempt might actually reduce the benefit of a subsequent kyphoplasty and cause sustained postural disadvantages to the patient as fracture consolidation hinders LKA restoration. However, there is no consistent data available yet regarding the influence of surgical timing on vertebral height restoration^20–22^.

Apart from the radiographic benefits of the operation, we also observed a significant pain reduction in our patients postoperatively. This is in keeping with existing studies on the short-term clinical outcome after kyphoplasty^6,7^. However, this observation might also raise suspicion regarding the origin of the postoperative SVA improvement. It seems plausible that pain relief by itself might lead to a more upright posture, even in the absence of a local height restoration of the fractured vertebra. In this case, one would have to suspect the effect of kyphoplasty on posture to be temporary as the acute fracture-related pain often fades over time under conservative treatment as well. Therefore, we examined for the first time whether the global postoperative changes of spinal alignment correlate rather with postoperative pain relief or vertebral body height restoration. Our results demonstrate that postoperative improvement in SVA correlates significantly with the correction of the LKA, but not with postoperative pain relief. This is remarkable, as it might indicate a long-lasting advantage of kyphoplasty over conservative treatment. However, it should be noted that the patients included in our study had (on average) only mild to moderate pain with an average VAS score of 3.7 at rest and 5.1 in motion. A stronger relationship between VAS and SVA might be conceivable in other populations where patients have stronger preoperative pain and therefore a more pronounced pain-related spinal malposition preoperatively. Theoretically, it is therefore possible that there might be an even better restoration of the sagittal profile in patients with stronger preoperative pain than in our cohort. However, this does not influence the correlation between LKA restoration and SVA improvement observed in our study independently from pain relief. Those to profit particularly from the procedure seem to be patients with a severely compromised posture preoperatively as the mean postoperative improvement in SVA was more than twice as high in patients with a positive sagittal imbalance preoperatively as compared to the general cohort.

Despite our efforts for a rigorous methodology to ensure reliable results, there are limitations to our study and the results should be interpreted accordingly. As the study relies on the assessment of retrospectively gathered patients treated with kyphoplasty, it is not possibly to precisely quantify the actual advantage over non-surgical treatment. Prospective studies will be needed to evaluate the long-term effect of the restoration of the sagittal profile on quality of life. Yet, existing literature indicates that the adverse effects of vertebral compression fractures on the quality of life, physical function, mental health, and life-span are related to the severity of the spinal deformity and partly independent of pain, which suggests a significant benefit from a restoration of the sagittal profile^21,23,24^. Finally, we performed multiple testing using data from the same patients, which may result in a risk of erroneous inferences. We this issue in our statistical analysis by rigorously adjusting the p-values according to the established Bonferroni-Holm method.

As a conclusion, we think that our data provide important new points to consider when deciding upon conservative vs. operative treatment in patients with vertebral fractures. While guidelines mainly recommend to consider kyphoplasty only after a futile conservative treatment attempt, treating physicians should also keep in mind that patients (especially those with a sagittal imbalance) might benefit from a restored posture irrespective of pain relief and that this might only be possible for a certain time after fracture occurrence.
